# Dr. James Evelyn Pilcher

**Published:** 1911-05

**Authors:** 


Dr. James Evelyn Pilcher, major in the medical department of the United States Army, (retired) died at his home at Carlisle, Pa., April 9, 1911, aged 54 years. Dr. Pilcher was well known in Buffalo where he had many friends. For many years he was secretary of the Association of Military Surgeons and editor of *The Military Surgeon* up to two years ago, when failing health made his retirement from active editorial management of the journal compulsory. Dr. Pilcher was graduated from the Long Island College Hospital, Brooklyn, in 1880, and three years later entered the medical corps of the army. Five years later he was promoted to be captain. During the Spanish-American war he served as major and brigade surgeon of volunteers and was retired in 1900 for disability incurred in the line of duty. He made Carlisle, Pa., his home and remained there with the exception of a few months spent in Richmond, Va., up to the time of his death. He was president and secretary of the Cumberland Valley Medical Association and a member of the American Medical Association.

